# Synthesis and characterization of silica xerogel from corn husk waste as cationic dyes adsorbent

**DOI:** 10.12688/f1000research.75979.1

**Published:** 2022-03-11

**Authors:** Ayu Dahliyanti, Devi Aryanihanan Yunitama, Iftahul Maulina Rofiqoh, Mazli Mustapha

**Affiliations:** 1 Department of Chemical Engineering, Universitas Pertamina, Jakarta, 12220, Indonesia; 2 Department of Mechanical Engineering, Universiti Teknologi PETRONAS, Seri Iskandar, 32610, Malaysia

**Keywords:** corn husk, silica xerogel, sol-gel method, cationic dyes, adsorption

## Abstract

**Background:** High corn production and consumption in Indonesia have led to massive corn husk waste. To increase the economic value of corn husk waste, innovations have utilized its silica content as an alternative source for the synthesis of multifunctional silica compounds.

**Methods:** In this research, silica xerogel was successfully synthesized from corn husk via the sol-gel method. Its physical properties and capabilities as an adsorbent to remove cationic dyes such as crystal violet and methylene blue in wastewater were investigated for the first time.

**Results:** The as-synthesized silica xerogel possesses an amorphous crystal structure with an average pore diameter of 6.35 nm, a Brunauer, Emmett, and Teller (BET) surface area of 363.72 m ^2^/g, and particle size ranging from 80 to 250 μm. The optimum contact times of silica xerogel are 90 minutes for crystal violet and 120 minutes for methylene blue adsorptions. While at the same time, the dye removal efficiencies are as high as 98.539% and 96.184%, respectively. The adsorption of both crystal violet and methylene blue on silica xerogel follows the Langmuir isotherm model and exhibits a high monolayer capacity of 69.44 mg/g and 59.17 mg/g, respectively.

**Conclusions: **These findings suggest the great potential of silica xerogel synthesized from corn husk as a low-cost and effective cationic dye adsorbent.

## Introduction

 Corn ( *Zea mays* L.) is one of the primary food commodities in Indonesia. In 2018, total corn production reached 30 million tons and increased by 308 thousand tons in 2019 ^1^. Food production mainly utilizes the various parts of the corn kernel only ^2^. Consequently, a massive amount of waste is generated from the rest of the corn plant such as the husk, cob, and stalk, which is estimated to reach 87.5 million tons a year ^3^. This waste is mainly used as animal feed, traditional cigarette wrappers, and handicraft materials with low economic value.

Based on previous research, the corn husk has the highest silica content compared to other parts of the corn including the stalk, cob, and leaves ^4^. The silica content has been extracted from corn husk ash via the leaching method with a yield of 2.543 wt%. However, the resulting silica powder from this method still contained some contaminants and a non-uniform crystal structure was produced ^4^. Another possible method of extracting silica is by using the sol-gel method, which yields silica xerogel as the final product. The sol-gel method possesses some benefits such as high product purity, high homogeneity, high surface area, and low extraction temperature ^5, 6^. 

Silica xerogel has a long history of application as a carrier, thickener, adsorbent, anti-caking, and free-flow agent ^6– 8^. A number of studies have also introduced silica xerogel as a cationic dye adsorbent. Even a low concentration of cationic dyes, such as methylene blue and crystal violet, in industrial effluents, can cause concern due to their harmful effects on human health and the aquatic ecosystem ^9^. Silica xerogel from bagasse ash and volcanic tuff adsorbed methylene blue with a maximum capacity of 23.3 and 51.97 mg/g ^10, 11^. Mesoporous silica, which was prepared through a wet chemical method from bagasse ash, was tested as a crystal violet adsorbent with a maximum capacity of 26.53 mg/g ^12^. The adsorption occurs mainly due to the electrostatic interaction between the positive charge of the cationic dye and the negative charge of the silanol (Si-OH) functional group on silica xerogel surface sites ^10, 11, 13^.

In this study, a novel silica xerogel adsorbent was synthesized from corn husk waste via the sol-gel method. Then, it was characterized by various methods to analyze the purity, crystallinity, morphology, particle size, surface area, and average pore diameter. The as-synthesized silica xerogel was then employed as an adsorbent to remove crystal violet and methylene blue. This proposed low-cost alternative could be applied to treat dye-containing wastewater generated from various industries while at the same time contributing to the reduction of agricultural waste.

## Methods

 Corn husk waste was sourced from cornfields in Jombang, East Java. Sodium hydroxide (99%), hydrochloric acid (37%), crystal violet, and methylene blue were purchased from Merck without any further purification.

### Preparation of silica xerogel from corn husk

 The dried corn husk, which was collected from July to December 2020, was burned to ash in a muffle furnace at 600°C for two hours at a heating rate of 10 °C/min to produce corn husk ash. Silica was extracted from 0.25 g of corn husk ash using 6 mL of 1 M sodium hydroxide. The mixture was stirred continuously at 80 °C for one hour to dissolve the silica. The mixture was cooled and centrifuged at 12,000 rpm for 10 minutes to remove the residue from the sodium silicate solution. The sodium silicate solution was neutralized with 3 M hydrochloric acid under constant stirring until it reached a pH of 7 to produce sol. The sol was aged at room temperature for 18 hours to promote hydrogel formation. After aging, the hydrogel was washed repeatedly using deionized water then centrifuged for five minutes at 4000 rpm. The hydrogel was dried at 60 °C for five hours to obtain silica xerogel. The yield of silica was calculated by the following equation:


$$Yield\;(\%)=\frac{mass\;of\;silica\;xerogel\;x\;100}{mass\;of\;corn\;husk\;ash}\;(1)$$

### Characterization of silica xerogel from corn husk

The identification of functional groups in the silica xerogel was conducted by Fourier transform infrared spectroscopy (FTIR, Thermo Scientific iS 5), scanned from 600 to 4000 cm ^-1^. The crystallinity of the xerogel was confirmed by X-ray diffraction (XRD, PANalytical) with Cu-Kα radiation and 2-theta ranging from 10–90°. The morphology, particle size, and elemental composition were examined using scanning electron microscope and energy dispersive X-ray spectroscopy (SEM-EDS, Desktop Phenom ProX). The Brunauer, Emmett, and Teller (BET) specific surface area as well as Barrett, Joyner, and Halenda (BJH) average pore diameters were measured by Micromeritics Tristar II 3020.

### Adsorption studies

The adsorption experiments were performed in a batch setup by adding 10 mg of silica xerogel to 10 ml of cationic dyes (crystal violet and methylene blue) at the initial concentrations of 10, 20, 30, 40, and 50 mg/L at 25°C for 30, 60, 90, 120, and 150 minutes. The final concentration of the solution was analyzed using a UV-Vis spectrophotometer by measuring absorbance at *λ* = 664 nm for methylene blue and *λ* = 590 nm for crystal violet. The following equation was used to calculate the adsorption capacity:


$$\;q_{e}=\frac{(C_{o}-C_{e})\;x\;V}{W}\;(2)$$

The following equation was used to calculate the removal efficiency (%) of dyes:


$$Removal\;efficiency\;(\%)=\frac{(C_{o}-C_{e})\;x\;100}{C_{o}}\;(3)$$

where C _o_ and C _e_ (mg L ^-1^) represent the initial and final concentration of crystal violet and methylene blue, w (g) is the mass of silica xerogel, and V (L) is the volume of solution. Then, the data were fitted against the Langmuir and Freundlich isotherm model based on the following equations:


$$Langmuir:\frac{C_{e}}{q_{e}}=\frac{C_{e}}{q_{max}}+\frac{1}{K_{L}\cdot q_{max}}\;(4)$$

$$Freundlich:log\;q_{e}=\frac{1}{n}log\;C_{e}+log\;K_{F}\;(5)$$

## Results and discussion

### Preparation of silica xerogel from corn husk

**Figure 1.**
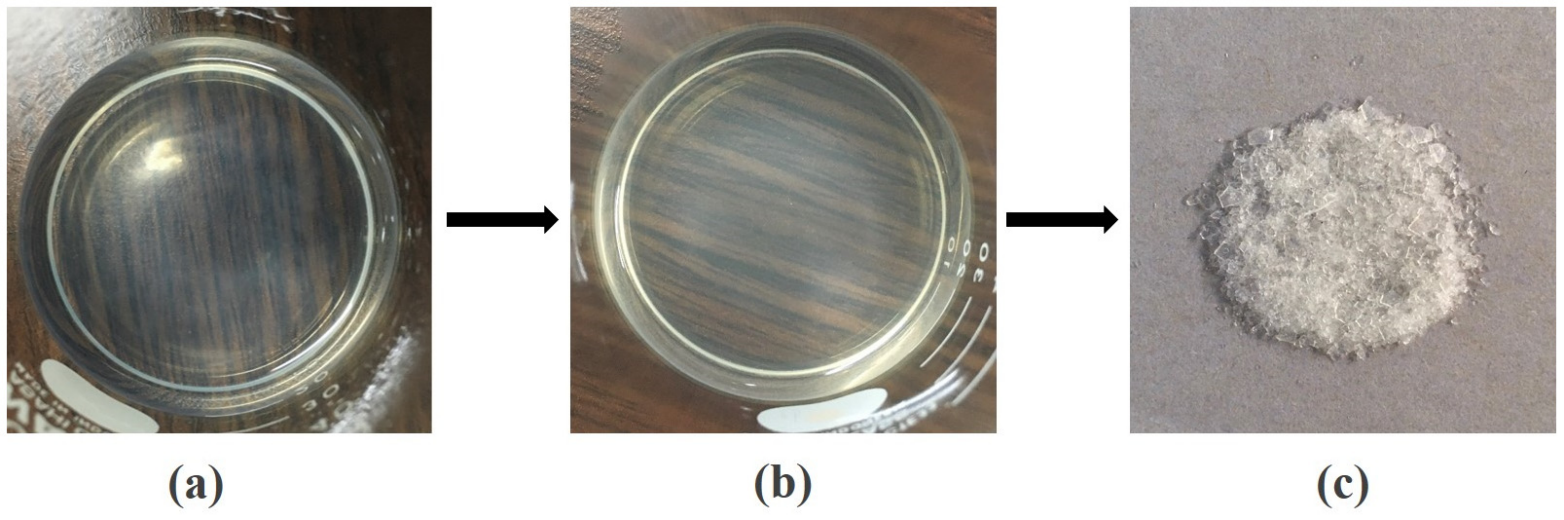
Transformation of ( **a**) sol ( **b**) hydrogel and ( **c**) xerogel via the sol-gel method.

The transformation of corn husk ash to hydrogel and xerogel by the sol-gel method is shown in Figure 1.

In the preparation of silica xerogel, sol was formed at sodium silicate acidification when hydrochloric acid was added to sodium silicate until pH=7 was obtained, as shown in Figure 1(a). The acidification reaction was then followed by the creation of a siloxane linkage between silanol groups on surface sites ^14^. This resulted in the formation of the hydrogel as displayed in Figure 1(b). The hydrogel was dried to form xerogel in solid form as shown in Figure 1(c) with a maximum yield of 22.457%.

### Characterization of silica xerogel

**Figure 2.**
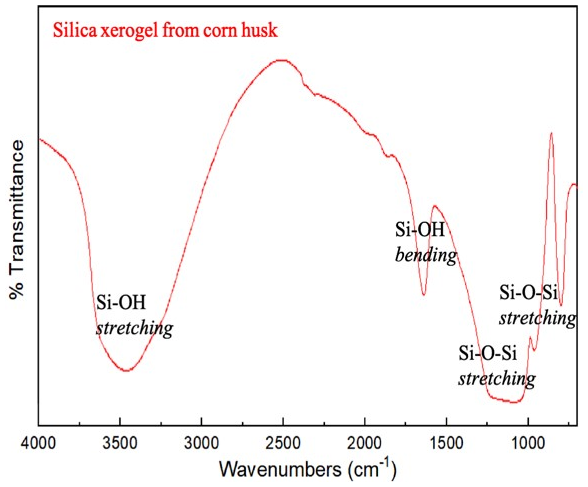
Fourier transform infrared spectroscopy (FTIR) spectrum of silica xerogel prepared from corn husk.

The FTIR spectrum of silica xerogel from corn husk is shown in Figure 2 ^15^. The peaks at 1650 cm ^-1 ^and 3500 cm ^-1^ are associated with symmetric bending vibration and asymmetric stretching vibration of Si-OH, respectively. Meanwhile, peaks at 1100 and 800 cm ^-1^ correspond to the Si-O stretching vibration in Si-O-Si bonds. ^6, 10^. Thus, we can conclude that silica xerogel has been successfully synthesized from corn husk waste.

**Figure 3.**
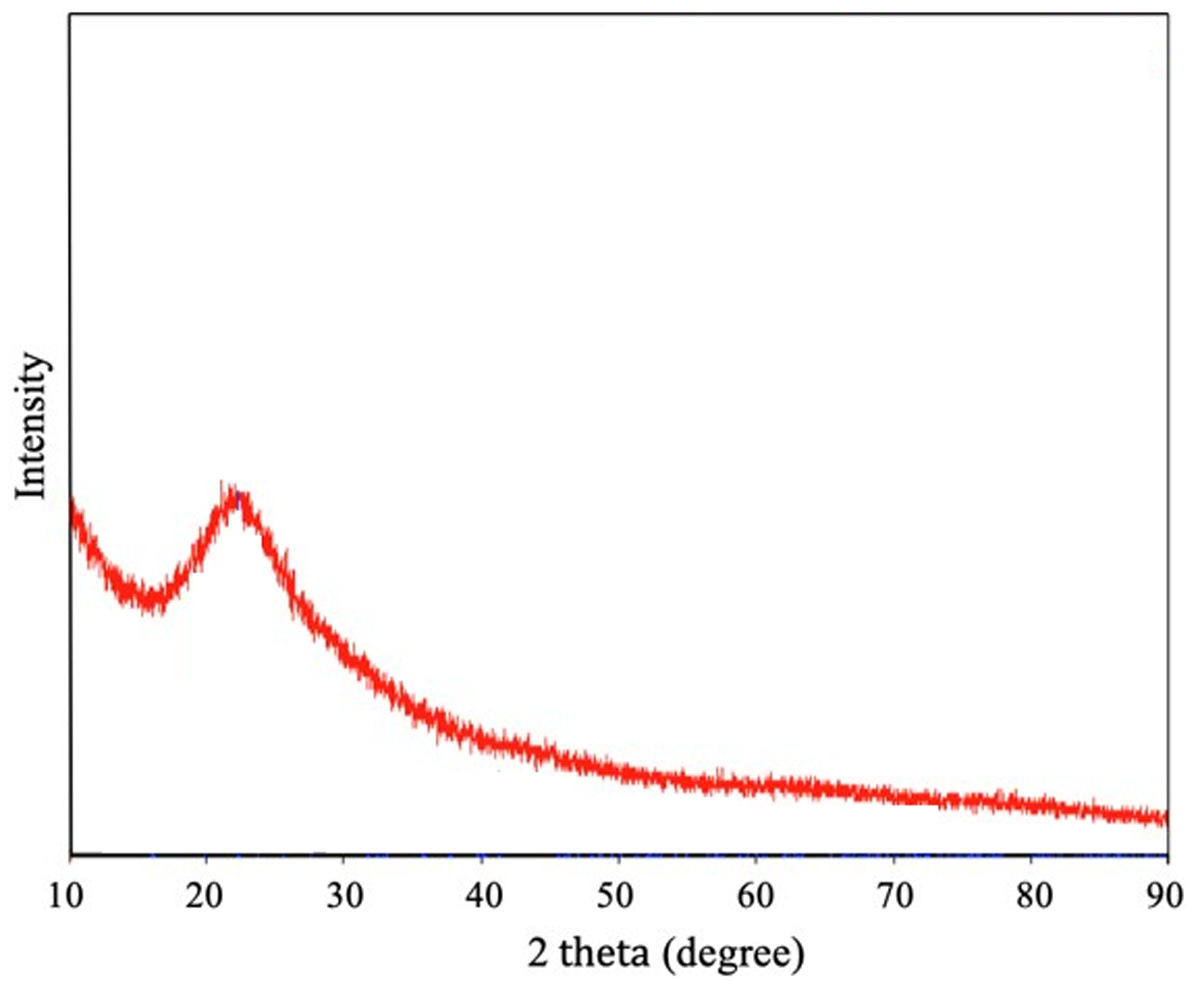
X-ray diffractograms of silica xerogel from corn husk.

X-ray diffraction patterns of silica xerogel in the range of 2-theta 10–90° are shown in Figure 3 ^16^. A broad peak at 2-theta of 17–28° indicates the characteristic amorphous silica structure. No other peak was detected implying the sample has high purity.

**Figure 4.**
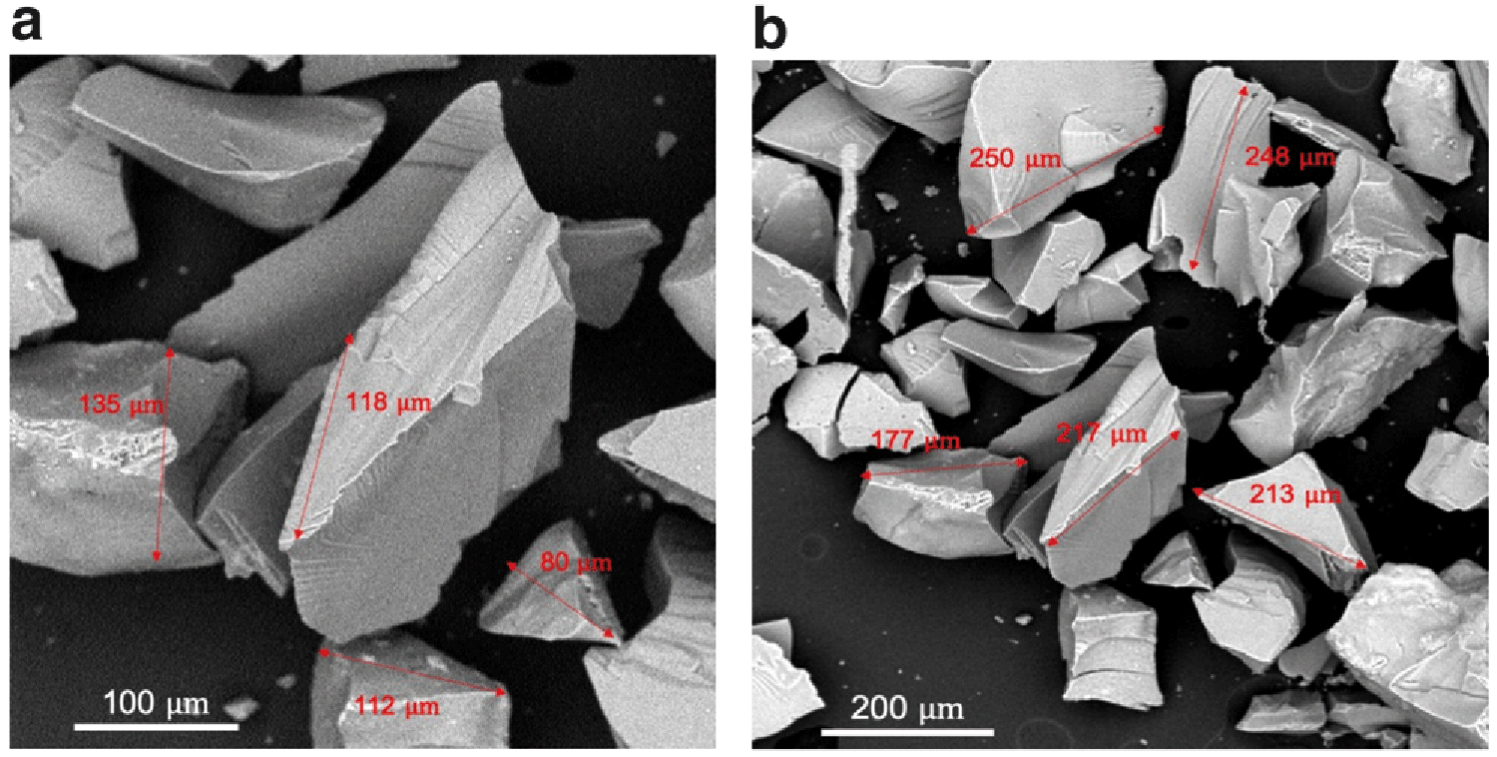
Scanning electron micrographs of silica xerogel from corn husk.

SEM micrographs of silica xerogel are displayed in Figure 4 ^17^. The morphology of the obtained particles gives some resemblance to flakes or shards with sizes ranging from 80–250 μm.

**Figure 5.**
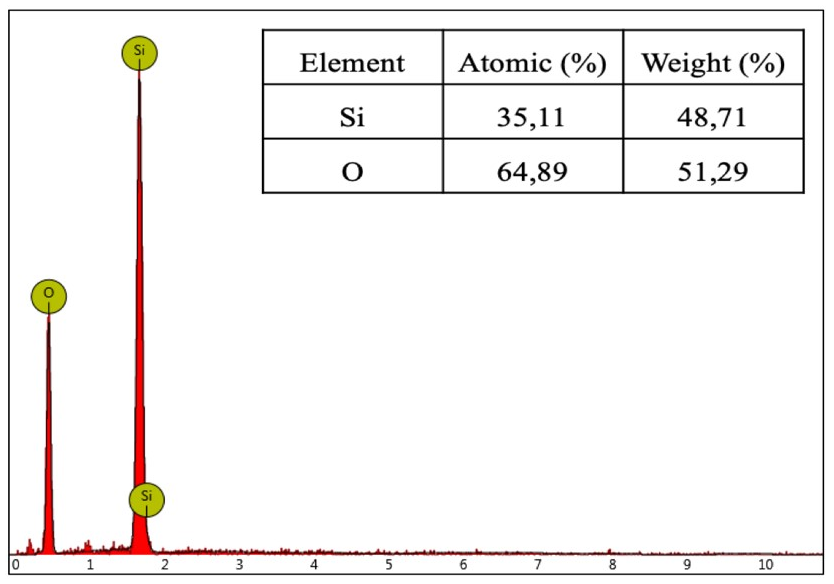
Energy dispersive X-ray spectra and composition of silica xerogel from corn husk.

The EDS spectrum of silica xerogel in Figure 5 shows the high purity of the silica xerogel, which has silicon and oxygen as the main constituents without any other impurities ^17^.

**Figure 6.**
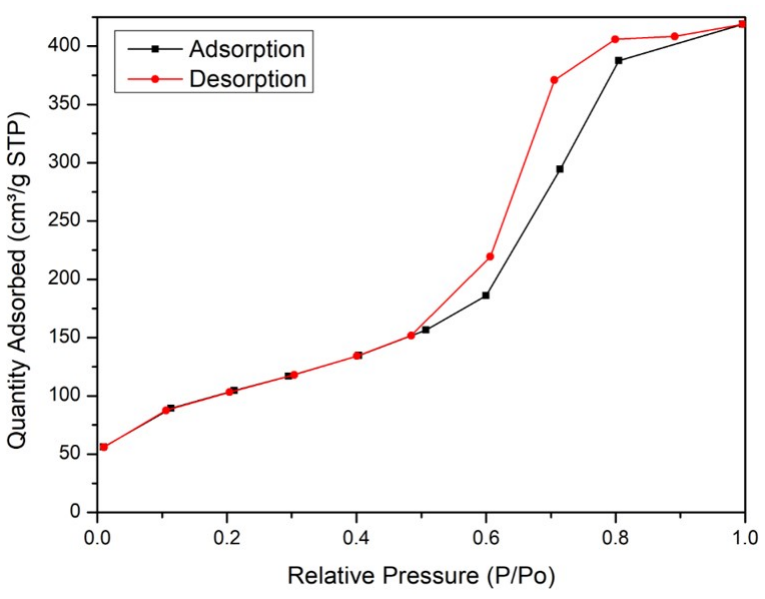
N _2_ adsorption-desorption isotherms of silica xerogel from corn husk.

The N _2_ adsorption-desorption isotherms of silica xerogel from corn husk are displayed in Figure 6 ^18^. According to IUPAC classification, the adsorption isotherm of as-synthesized silica xerogel belongs to the type-IV, which is typical of mesoporous material with size ranging from 2 to 50 nm. The BET surface area and the BJH average pore diameters are 363.72 m ^2^/g and 6.35 nm, respectively.

### Adsorption studies

**Figure 7.**
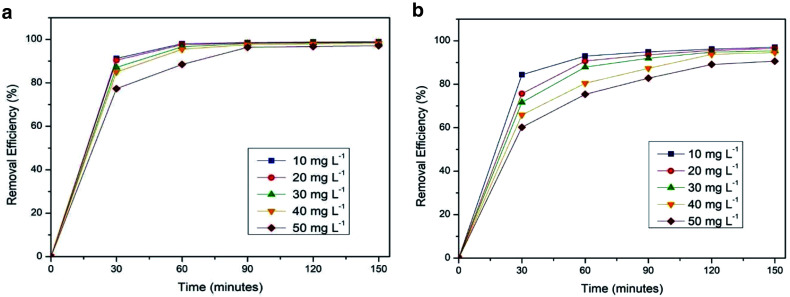
The effect of contact time on the removal efficiency of ( **a**) crystal violet and ( **b**) methylene blue.

The relationship between contact time and the adsorption of both cationic dyes at different initial concentrations is shown in Figure 7 ^19, 20^. In general, the removal efficiency of silica xerogel for crystal violet and methylene blue increases with prolonged contact time. However, the adsorption rate becomes slower as it approaches equilibrium. This may be caused by the unavailability of active sites on the adsorbent surface after a certain period ^21, 22^.

 As shown in Figure 7(a), the optimum adsorption condition for crystal violet is obtained at a contact time of 90 minutes with a removal efficiency of 98.539%. Meanwhile, as displayed in Figure 7(b) the optimum adsorption condition for methylene blue is obtained at a contact time of 120 minutes with a removal efficiency of 96.184%. 

From the calculated correlation coefficients (R ^2^) in Table 1 ^19, 20^, the adsorption process of both dyes on silica xerogel is a good fit with the Langmuir isotherm model. This suggests that the adsorption process is inclined toward monolayer chemisorption due to electrostatic interactions between the positively charged cationic dyes molecule and negatively charged surface sites ^23, 24^.

**Table 1.** Adsorption isotherm parameters of crystal violet and methylene blue on silica xerogel from corn husk (T = 25°C and P = 1 atm).

Models	Parameters	Adsorbate	
		Crystal violet	Methylene blue
Langmuir	q _max_ (mg/g)	69.44	59.17
	K _L_ (L/mg)	1.2	0.6
	R ^2^	0.996	0.996
Freundlich	K _F_ (mg/g)	36.8	19.28
	n	1.6	1.7
	R ^2^	0.969	0.973

The maximum adsorption capacity (q _max_) for crystal violet is as high as 69.44 mg/g and 59.17 mg/g for methylene blue. Compared to other crystal violet adsorbents from previous research, silica xerogel from corn husk has a higher maximum adsorption capacity ^13, 24– 26^. The as-synthesized silica xerogel also performed better in methylene blue adsorption compared to other adsorbents ^11, 27– 29^. The high surface area and suitable pore diameter of silica xerogel arguably also contributes to the favorable interactions between the adsorbate molecules and the adsorbent surface.

## Conclusions 

 Silica xerogel with high purity was successfully synthesized from corn husk waste via the sol-gel method with a maximum yield of 22.457 wt% of corn husk ash. Silica xerogel had an amorphous phase with mesoporous and irregular shapes. The as-synthesized silica xerogel performed better than many other cationic dyes adsorbents in removing crystal violet and methylene blue, which was proven by its high adsorption capacity. Silica xerogel from corn husk is a good adsorbent for cationic dyes because it has a high surface area and small pore diameter. This research is still in its early stages, and we need to examine the effect of other parameters such as particle size, pH, and temperature to improve the adsorption capabilities of silica xerogel from corn husk. Nonetheless, these results can be used as a basis for developing it further into a continuous process that can be applied for industrial wastewater treatment.

## Data availability

### Underlying data

Figshare: Underlying data for ‘Synthesis and characterization of silica xerogel from corn husk waste as cationic dyes adsorbent’.

The following underlying data is provided:

- FTIR spectrum of silica xerogel synthesized from corn husk via the sol-gel method, https://doi.org/10.6084/m9.figshare.17073707.v1 ^15^
- XRD data of silica xerogel synthesized from corn husk via the sol-gel method, https://doi.org/10.6084/m9.figshare.17073785.v1 ^16^
- SEM and EDS data of silica xerogel synthesized from corn husk via the sol-gel method, https://doi.org/10.6084/m9.figshare.17073980.v1 ^17^
- BET data of silica xerogel synthesized from corn husk via the sol-gel method, https://doi.org/10.6084/m9.figshare.17073848.v2 ^18^
- Adsorption experiments data of crystal violet on silica xerogel, https://doi.org/10.6084/m9.figshare.17074247.v1 ^19^
- Adsorption experiments data of methylene blue on silica xerogel, https://doi.org/10.6084/m9.figshare.17074325.v1 ^20^

Data are available under the terms of the Creative Commons Attribution 4.0 International license (CC-BY 4.0).
