# Supplementary material for: Synthesis and characterization of silica xerogel from corn husk waste as cationic dyes adsorbent
Source: F1000Res. 2022 Mar 11;11:305. [Version 1] doi: 10.12688/f1000research.75979.1 (PMC9372640; doi:10.12688/f1000research.75979.1)
Supplement: Peer review history

# Peer review: Reviewer response for version 1

**DOI:** 10.5256/f1000research.79927.r145519

**Published:** 2022-08-11

**Authors:** Witta Kartika Restu

**Affiliations:** 1 Indonesian Institutes of Sciences (LIPI), South Tangerang, Indonesia

I have read carefully the article entitled “Synthesis and characterization of silica xerogel from corn husk waste as cationic dyes adsorbent”. The study is interesting because it utilized the waste derived from natural resources as the source for the xerogel that acts as the adsorbent. Even though it was sourced from the waste, the silica xerogels have high removal efficiencies for the dyes, around 96-98%. However, this will be improved if the silica xerogel can have faster optimum contact time with the cationic dyes. Therefore, this present study needs further research to investigate other parameters, such as the particle size, pore diameter and surface interaction, in order to achieve the best performance with the minimum contact time for the dye removal.

Is the work clearly and accurately presented and does it cite the current literature?

Yes

If applicable, is the statistical analysis and its interpretation appropriate?

Not applicable

Are all the source data underlying the results available to ensure full reproducibility?

Yes

Is the study design appropriate and is the work technically sound?

Yes

Are the conclusions drawn adequately supported by the results?

Yes

Are sufficient details of methods and analysis provided to allow replication by others?

Yes

Reviewer Expertise:

Macromolecules chemistry, Biomaterial, Biopolymer

I confirm that I have read this submission and believe that I have an appropriate level of expertise to confirm that it is of an acceptable scientific standard.

# Peer review: Reviewer response for version 1

**DOI:** 10.5256/f1000research.79927.r127450

**Published:** 2022-04-05

**Authors:** K. Rajeev Kumar

**Affiliations:** 1 Department of Instrumentation, Centre of Excellence in Advanced Materials, Cochin University of Science and Technology, Cochin, Kerala, India

Report of the review of the paper titled ‘Synthesis and characterization of silica xerogel from corn husk waste as cationic dyes adsorbent’.

Note: Sol-gel method of synthesis of silica xerogel from corn husk ash is not reviewed.

 The paper describes the production of silica xerogel from corn husk ash by sol-gel method. The reported relevance of the present method is to increase the economic value of corn husk waste. The silica xerogel thus produced is used as an adsorbent to remove cationic dyes such as crystal violet and methylene blue in wastewater. Even though the authors claim that this is the first-time silica xerogel is used for such purposes, a search reveals other reports also.

 The produced silica xerogel is subjected to different characterization techniques such as UV-Vis spectroscopy, FTIR, XRD, EDAX, SEM and BET surface area & BJH average pore size measurements. All measurements are relevant and useful to identify the material, chemical composition, morphology, surface area and pore size. However, the UV-Vis spectrum is not given in the paper. (Data shown as ‘partly’ in the objective part of the report indicates the absence of the UV-Vis absorption data). It is advisable to add UV-Vis adsorption spectrum also. Removal efficiencies for crystal violet and methylene blue are 98.539% and 96.184% which are quite impressive and better than most of the reported values for same type of work.

**Recommended for indexing.**

Is the work clearly and accurately presented and does it cite the current literature?

Yes

If applicable, is the statistical analysis and its interpretation appropriate?

Not applicable

Are all the source data underlying the results available to ensure full reproducibility?

Partly

Is the study design appropriate and is the work technically sound?

Yes

Are the conclusions drawn adequately supported by the results?

Yes

Are sufficient details of methods and analysis provided to allow replication by others?

Yes

Reviewer Expertise:

Material Science, Atomic Layer Deposition, Transition Metal Dichalkogenides (TMDs)

I confirm that I have read this submission and believe that I have an appropriate level of expertise to confirm that it is of an acceptable scientific standard.
